# Double-Expressor Appendiceal Burkitt's Lymphoma: A Case Report and Literature Review

**DOI:** 10.1155/2022/6795699

**Published:** 2022-03-25

**Authors:** Osama N. Dukmak, Hamzeh M. I. Abugharbieh, Mohammad Farid Emar, Iman Khamayseh, Salem M. Tos, Rafiq Salhab

**Affiliations:** ^1^Al-Quds University, Faculty of Medicine, Jerusalem, State of Palestine; ^2^Al-Ahli Hospital, Hebron, State of Palestine

## Abstract

**Background:**

Appendiceal lymphoma is a very rare entity accounting for 0.015% of all gastrointestinal lymphoma cases. Acute appendicitis is the most common presentation of primary appendix neoplasms. Burkitt's lymphoma presenting as an acute appendicitis is a rare entity with around 21% of the cases presenting as a lower iliac fossa mass. *Case Presentation*. A 23-year-old male was admitted to the surgical ward as a case of acute appendicitis with localized tenderness in the right iliac fossa, positive rebound tenderness, a positive Rovsing's sign, and ultrasound findings of suspected complicated appendicitis. Appendectomy was performed. Histopathological examination of the appendectomy specimen revealed a double-expressor non-Hodgkin diffuse large cell lymphoma with Burkitt's-like morphology. He was sent for chemotherapy treatment.

**Conclusion:**

Only 34 cases of Burkitt's lymphoma have been reported to present as acute appendicitis. Histological examination following appendectomy for an apparent appendicitis is essential. Furthermore, complete blood count and a computed tomography scan aid the diagnosis of lymphoma. Double-expressor lymphoma has been shown to have poor outcomes. Therefore, prompt and aggressive treatment is vital.

## 1. Introduction

Neoplasms of the appendix are rare and account for only 0.5 to 1 percent of intestinal neoplasms [1] and found in ∼0.5–1.0% of appendectomy specimens at pathologic evaluation [2]. Appendiceal lymphomas are exceedingly rare and constitute around 0.015% of all gastrointestinal lymphoma cases [2].

Burkitt's lymphoma (BL) is a highly aggressive B-cell non-Hodgkin lymphoma characterized by the translocation and deregulation of the Myc gene on chromosome 8. A case series of 116 patients with appendiceal lymphomas showed that BL was the second most prevalent pathology, diagnosed in 25.9% of patients [2].

This rapidly growing tumor can cause symptoms due to mass effect or direct involvement of the bowel [3] which may manifest as bowel obstruction, intussusception, or appendicitis [4].

Acute appendicitis is the most common presentation of primary appendix neoplasms [5]. Therefore, histological examination following appendectomy for an apparent appendicitis is essential and can provide the diagnosis of BL, which leads to the specific disease management. Herein, we present a 23-year-old male patient who presented with acute appendicitis and was found to have BL.

## 2. Case Presentation

A 23-year-old male presented to our center complaining of right lower quadrant (RLQ) abdominal pain of 12-hour duration.

The pain started in the periumbilical region and then became more localized to the right iliac fossa where the pain was colicky in nature and was increasing in severity. Moreover, the pain was not related to food or movement.

Furthermore, the patient had nausea and vomiting. However, the patient denied any change in bowel habits or any history of dysuria or change of urine color.

On physical examination, the patient had normal vital signs. The abdomen was normal in shape with a normal inverted umbilicus. There were no visible scars or dilated veins. Moreover, the abdomen was soft with localized tenderness in the right iliac fossa and positive rebound tenderness. Rovsing's sign was also positive. However, there were no features of generalized peritonitis and no masses or organomegaly.

The patient had a sore throat with enlarged cervical lymph nodes 1 week ago and was managed as acute tonsillitis.

Abdominal ultrasound revealed complicated appendicitis.

Urinalysis was normal and laboratory investigations were as follows: hemoglobin level was 16.25 gm/dl (N: 13.5 to 17.5 gm/dl for men); RBC count was 5.663 mCL (N: 4.6–6.2 mCL); WBC was 14.07 × 10^3^ (N: 4–11 × 10^3^ uL); neutrophils were 70.4% (N: 45–65%); platelets count was 99.39 × 10^9^ L (N: 150–400 × 10^9^ L); lymphocyte was 3.76% (N: 25–45%); monocyte was 19.3% (N: 0–6%); basophil was 6.51% (N: 0–1%).

The patient was found to have positive Epstein-Barr virus (EBV) IgG antibodies but negative EBV IgM antibodies. Cytomegalovirus antibodies (CMV-IgG and CMV-IgM) were also negative.

**Figure 1:**
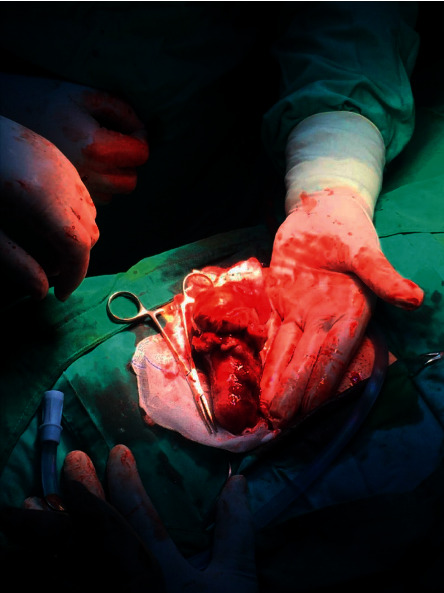
The appendiceal mass found intraoperatively.

We performed an appendectomy through a McBurney's abdominal incision and found a perforated huge appendiceal mass with mild to moderate fluid (Figure 1); the base of the appendix was normal. Free abdominal fluid was not aspirated. The remainder of the intra-abdominal organs appeared unremarkable.

Postoperatively, the patient was sent to the surgical ward and was kept NPO with IV fluids and IV antibiotics. The patient was afebrile and had stable vital signs. Hospital stay was three days with no postoperative complications.

**Figure 2:**
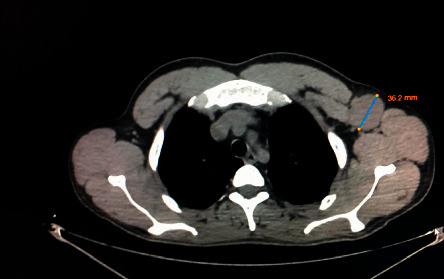
CT scan postoperatively showing a soft tissue lesion measuring 36.2 mm in the left axilla consistent of lymph node involvement.

Whole-body CT scan postoperatively showed a soft tissue lesion in the left axilla consistent with lymph node involvement (Figure 2). CT scan also showed liver enlargement (about 20 cm) but no focal lesions. However, there was no mesenteric lymphadenopathy.

**Figure 3:**
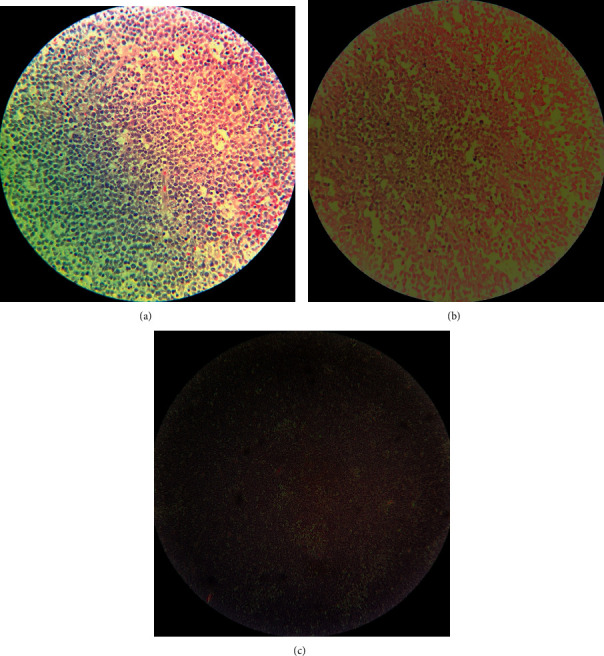
Histopathological examination of the appendectomy specimen. (a) Proliferation of poorly cohesive malignant lymphoid cells, composed of medium-sized to large nuclei, multiple prominent nucleoli, and scant cytoplasm. (b) Tumor cell necrosis. (c) Starry sky appearance.

Histopathological examination of the appendectomy specimen revealed a non-Hodgkin diffuse large cell lymphoma with Burkitt's-like morphology. Specifically, the hematoxylin and eosin slide demonstrated sheets of highly neoplastic lymphoblasts with high nucleus-to-cytoplasmic (N : C) ratio, hyperchromasia, and apoptotic bodies giving focally starry sky appearance. Zones of necrosis and brisk mitosis were also identified (Figures 3(a)–3(c)).

Immunohistochemistry testing demonstrated the cells being positive for CD20, CD79a, CD10, CD3, Bcl-2, Bcl-6, C-Myc (>40%), and Ki-67 (expressed in >95% of lymphoma cells) and negative for CD30 and CD43.

Genetic testing and FISH were not done unfortunately because they are unavailable at our hospital.

The patient was diagnosed with double-expressor lymphoma according to the immunohistochemistry testing (co-expression of Myc and Bcl2 proteins).

Histology for specimens taken from the bone marrow and cerebrospinal fluid (CSF) showed no evidence of tissue involvement with lymphoma.

The patient thereafter was started on chemotherapy with four cycles of R-IVAC (rituximab, ifosfamide, etoposide, cytarabine), mesna, neupogen, cytarabine, methotrexate, and folic acid. His chemotherapy treatment was complicated with mild anemia, mild thrombocytopenia, nausea, and vomiting.

A PET scan done 7 months later showed no evidence of active lymphoma. The patient is currently alive and has been clear from lymphoma for 2 years.

## 3. Discussion

Acute appendicitis (AA) is traditionally a clinical diagnosis, but the diagnosis is further supported by laboratory and radiological investigations, such as ultrasound and CT scans [6]. Burkitt's lymphoma is further subdivided into three subtypes (endemic, sporadic, and immunodeficiency-associated) which vary in epidemiology, clinical presentation, and risk factors [7]. Sporadic subtype is associated with Epstein-Barr virus (EBV) with the most common site of involvement being the abdomen and commonly affecting the bowel [7]. Our patient had a positive EBV IgG and appendiceal tumor making the sporadic subtype of Burkitt's lymphoma more likely.

A diagnosis of Burkitt's lymphoma is dependent on a combination of histologic (diffuse lymphoid infiltration with scattered macrophages), immunophenotypic (CD20, CD10, Bcl6 positive, and Ki-67 near 100%), and genetic features (c-Myc translocation) [8]. All of these features were identified in the present case. Although the most common finding on flow cytometry is an expression of IgM immunoglobulin on the surface of biopsied tissue, mature B-cell markers such as CD19, CD20, CD22, CD79a, and CD10 can be found. However, CD5, CD23, CD34, and tdT are usually negative [9].

Patients with Burkitt's lymphoma who have an abdominal mass may present with nausea, vomiting, loss of appetite, gastrointestinal bleeding, signs and symptoms of acute abdomen, intestinal perforation, or renal failure [9].

Although preoperative CT scans can be used in confirming the presence of appendicitis with high sensitivity [6], they are not great at identifying if the cause is neoplastic [10]. A cohort study was published in 2020 which concluded that even if CT scans cannot identify neoplastic causes, they cannot exclude them either [10]. On the other hand, if the tumor has lymph node involvement, distant metastasis, or features that suggest an underlying malignancy, a preoperative CT scan could give us some hints on the underlying etiology [11]. Our patient did not have any abdominal lymph node involvement or metastasis. However, the patient's liver was enlarged which could indicate a malignancy. Therefore, an abdominal CT scan would give us a suspension but not a conformation. Our management would not have changed except for a more rapid histopathology report and aspirating free fluid in the abdomen in order to test for the presence of malignant cells.

In 2016, the WHO included a new category of lymphoma called high-grade B-cell lymphoma with translocations involving Myc gene and Bcl-6 or Bcl-2 genes. The lymphoma is termed a double hit if two rearrangements are present and triple hit if three rearrangements are present [12]. On the other hand, if the immunohistochemistry exhibits an expression of both Bcl2 and Myc proteins and not related to chromosomal rearrangement, then it is called double-expressor lymphoma [13].

Our patient has positive Myc and Bcl2 making the diagnosis of double-expression diffuse large B-cell lymphoma most likely.

Double-expressor lymphoma has worse outcomes than non-double-expresser lymphoma [14]. Double-expressor lymphoma is standardly treated by R-CHOP chemotherapy which includes rituximab, cyclophosphamide, doxorubicin, vincristine, and prednisone.

Even though there are several induction regimens for diffuse large B-cell lymphoma, patients who received R-HyperCVAD/MA (rituximab, cyclophosphamide, doxorubicin, vincristine, cytarabine, dexamethasone/methotrexate) and DA EPOCH-R (rituximab, cyclophosphamide, methotrexate/ifosfamide, doxorubicin, vincristine, etoposide, cytarabine) had higher rates of complete remission compared to R-CHOP [15].

In 2018, a retrospective study by Ichiro Kawashima and Yoshihiro Inamoto revealed that poor outcomes were noticed after allogeneic transplantation for double-expressor lymphoma [16].

The prognosis of lymphoma is dependent on many factors, such as the primary site of the lymphoma [17]. It has been shown that the 5-year survival rate of lymphomas in the ileocecal region was found to be 64.3% which is higher than that in large intestine (48.8%) and small intestine (32.5%) [17]. Primary central nervous system lymphoma on the other hand showed a low 5-year survival rate of 18% [18]. That being said, appendicular lymphomas have a good prognosis as it was shown that low-grade tumors including lymphomas have a 5-year survival rate of 67%–97% [19].

Another factor that plays a vital role in the prognosis of lymphoma is the histological type [20]. It was found that the 5-year survival rate of double-expressor lymphomas is 33%, which is 5–13 times higher than that of non-double-expressor ones [20]. Similar to double-hit lymphomas, triple-hit lymphomas have worse outcomes than normal ones [21].

Different systems of staging have been used to stage Burkitt's lymphoma, and we would like to emphasize on Murphy's staging system which has emphasis on extranodal disease which also distinguishes CNS disease and bone marrow disease [22].

Including this case, 34 cases of Burkitt's lymphoma have been reported in the literature to present as an acute appendicitis (see Table 1).[5].

A cohort study by Alexander H. Mimery revealed that most of the patients with appendiceal lymphoma were predominantly males accounting for 66.7% of the cases and the average age of onset was around 20 years. A palpable right iliac fossa mass was only identified in around 21% of the cases. However, an appendectomy was done in 65% of the cases (22 out of 34 cases), while 8 of the cases ended up in right hemi-colectomy.

## 4. Conclusion

Burkitt's lymphoma, although rare, may present as an acute appendicitis. Complete blood count and a computed tomography scan aid the diagnosis of lymphoma. Moreover, immunohistochemistry testing helps in detecting double/triple-hit Burkitt's lymphoma which has worse prognosis.

Only 34 cases of Burkitt's lymphoma have been reported to present as an acute appendicitis.

## Figures and Tables

**Table 1:** Characteristics of Burkett's lymphomas mimicking appendicitis as reported in the literature.

Publication year	Sex	Age	Ethnicity	Presentation	WBC (count/mm^3^)	Lymph node involvement	Surgical procedure	Chemo-therapy	Follow-up
2022	M	23	NA	Right lower quadrant (RLQ) pain	14070	Left axillary	Appendectomy	Yes	24 months in remission
2021 [23]	M	18	Not available	Pelvic pain	17400	Not available (NA)	Appendectomy	Yes	30 months in remission
2021 [24]	M	15	NA	RLQ pain	NA	Para-aortic	Appendectomy	Yes	NA
2020 [25]	M	22	NA	RLQ pain	15500	Para-aortic	Appendectomy	Yes	7 months in remission
2020 [9]	F	6	NA	Right iliac fossa (RIF) pain	44600	No	Cecectomy	Yes	NA
2019 [26]	F	40	NA	RIF pain	12400	NA	Appendectomy	Yes	NA
2018 [27]	F	13	NA	Diffuse abdominal pain	15100	NA	Appendectomy and salpingo-oophorectomy	Yes	NA
2018 [28]	M	36	NA	RLQ pain	13200	NA	appendectomy	Yes	NA
2017 [9]	M	16	NA	NA	NA	NA	Appendectomy	NA	NA
2017 [29]	M	20	Middle-Eastern	NA	NA	NA	Appendectomy	NA	NA
2016 [30]	M	53	NA	Diffuse abdominal pain and left flank pain	NA	No	Appendectomy	Yes	NA
2016 [31]	F	27	NA	RLQ pian	NA	Peri-ileal and pericaecal	Right hemi-colectomy	NA	NA
2015 [32]	M	17	NA	RLQ pain	7300	Mesentric	Laparoscopic exploration without appendectomy	Yes	After 2 months, a significant reduction in tumor masses
2015 [33]	16	F	NA	NA	NA	No	Right hemi-colectomy and end-lateral ileotransversostomy	Yes	NA
2014 [34]	10	M	NA	Fatigue and RLQ pain	12800	No	Appendectomy	Yes	14 months in remission
2014 [34]	23	M	NA	Abdominal pain, vomiting, and diarrhea	11800	No	Appendectomy	Yes	17 months in remission
2014 [34]	24	F	NA	RLQ pain	NA	No	Appendectomy	Yes	18 months in remission
2014 [4]	13	F	NA	RIF pain	NA	Ileal	Appendectomy	Yes	96 months in remission
2014 [4]	18	F	NA	Cecal fistula	NA	Ileocecal	Ileocecal resection and end-to-end anastomosis of the ileum and ascending colon	Yes	104 months in remission
2013 [35]	4	M	NA	Abdominal pain	12700	Perirectal and mesenteric	Incision and drainage of abscess	Yes	24 months in remission
2012 [36]	14	M	Caucasian	Periumbilical pain	11500	No	Appendectomy	Yes	12 months in remission
2010 [37]	10	M	NA	Periumbilical pain	15800	No	Appendectomy	Yes	Recurrence after 7 months + died after 8 months
2010 [38]	14	M	Caucasian	RIF pain	15100	No	Right hemi-colectomy	Yes	NA
2010 [5]	49	M	NA	Gross haematuria and RLQ pain	10800	Mesenteric	Right hemi-colectomy	Yes	1 month in remission
2006 [9]	14	M	NA	NA	NA	NA	Right hemi-colectomy	NA	NA
2006 [39]	60	M	Caucasian	RLQ pain	NA	No	Appendectomy	Yes	NA
2002 [9] [40]	12	M	European	RLQ pain	NA	NA	Right hemi-colectomy	NA	NA
1996 [9]	22	M	NA	NA	NA	NA	NA	NA	NA
1993 [41]	17	M	NA	RLQ pain	NA	No	Cecectomy	Yes	NA
1990 [42]	3	F	Caucasian	RLQ pain	17100	No	Appendectomy	Yes	5.5 months in remission
1984 [43]	22	M	Malay	Right sided abdominal pain	15800	Serosal	Appendectomy	Yes	2 months in remission
1983 [44]	22	M	NA	Epigastric and periumbilical pain	18200	No	Appendectomy	NA	NA
1980 [45]	8	M	Caucasian	Diffuse abdominal pain	10300	No	Appendectomy	Yes	36 months in remission
1980 [45]	10	M	White	Lower abdominal pain	19800	Mesenteric	Appendectomy	Yes	NA
